# From Prions to Stress Granules: Defining the Compositional Features of Prion-Like Domains That Promote Different Types of Assemblies

**DOI:** 10.3390/ijms22031251

**Published:** 2021-01-27

**Authors:** Anastasia Fomicheva, Eric D. Ross

**Affiliations:** Department of Biochemistry and Molecular Biology, Colorado State University, Fort Collins, CO 80523, USA; Anastasia.Fomicheva@colostate.edu

**Keywords:** prion, prion-like domain, stress granules, liquid–liquid phase separation, low-complexity domains

## Abstract

Stress granules are ribonucleoprotein assemblies that form in response to cellular stress. Many of the RNA-binding proteins found in stress granule proteomes contain prion-like domains (PrLDs), which are low-complexity sequences that compositionally resemble yeast prion domains. Mutations in some of these PrLDs have been implicated in neurodegenerative diseases, including amyotrophic lateral sclerosis and frontotemporal dementia, and are associated with persistent stress granule accumulation. While both stress granules and prions are macromolecular assemblies, they differ in both their physical properties and complexity. Prion aggregates are highly stable homopolymeric solids, while stress granules are complex dynamic biomolecular condensates driven by multivalent homotypic and heterotypic interactions. Here, we use stress granules and yeast prions as a paradigm to examine how distinct sequence and compositional features of PrLDs contribute to different types of PrLD-containing assemblies.

## 1. Introduction

Prions are protein-based transmissible agents. Most known prions result from the structural conversion of proteins from a soluble form into insoluble amyloid aggregates [1]. Prions were initially identified as the agents responsible for mammalian transmissible spongiform encephalopathies [2], but potential prions have subsequently been identified in a range of organisms, including yeast [3], other fungi [4], plants [5], and bacteria [6].

Yeast prions in particular have emerged as a powerful model system for studying the causes and consequences of this type of protein aggregation. Approximately 10 amyloid-based prions have been identified in yeast [7,8]. For most prion proteins, only a limited portion of the protein, termed the prion domain, is necessary for prion formation [9]. The majority of yeast prion domains share similar amino acid compositions, including a striking enrichment of glutamine and asparagine residues [9]. Various prediction algorithms have been developed to identify proteins with prion-like domains (PrLDs), defined as domains that are compositionally similar to these yeast prion domains [10,11,12,13,14,15]. PrLDs are surprisingly common in eukaryotic proteomes. PLAAC [14], a widely used prion prediction method, identifies approximately 240 and 200 PrLD-containing proteins in the human and yeast proteomes, respectively [10,16].

Only a small fraction of PrLDs have thus far been demonstrated to form bona fide prions [10]. However, PrLDs have been linked to various other types of macromolecular assemblies. In vitro, PrLDs can adopt a variety of phase-separated states that include liquid-like assemblies, gels, glasses, and amyloid aggregates. Likewise, in vivo, various PrLDs have been implicated in the formation of assemblies ranging from mnemons, which are nontransmissible amyloid assemblies that act as a form of cellular memory [17,18,19,20], to complex, dynamic biomolecular condensates, such as stress granules [21,22].

Stress granules have attracted particular attention due to their links to amyotrophic lateral sclerosis (ALS) and other degenerative diseases. Stress granules are dynamic, reversible ribonucleoprotein (RNP) assemblies that are formed in response to various cellular stresses, including heat and oxidative stresses, oxygen starvation, and viral infection [23,24]. Many PrLDs are found in RNA-binding proteins that localize to stress granules [25]. Some of these PrLDs appear to directly promote the recruitment of their respective proteins to stress granules [22,26], while others seem to act as modulators of recruitment, potentially helping to maintain solubility and prevent irreversible phase transitions [27,28]. Disease-associated mutations within PrLDs of stress granule proteins, such as FUS [29,30], TDP-43 [31,32], hnRNPA1 [33], and hnRNPA2B1 [33], are associated with the formation of persistent cytoplasmic inclusions that overlap in composition with stress granules. Based on these observations, it has been proposed that PrLDs promote stress granule formation and/or modulate stress granule dynamics, while disease-associated mutations perturb stress granule dynamics, leading to altered function [16,34].

**Figure 1:**
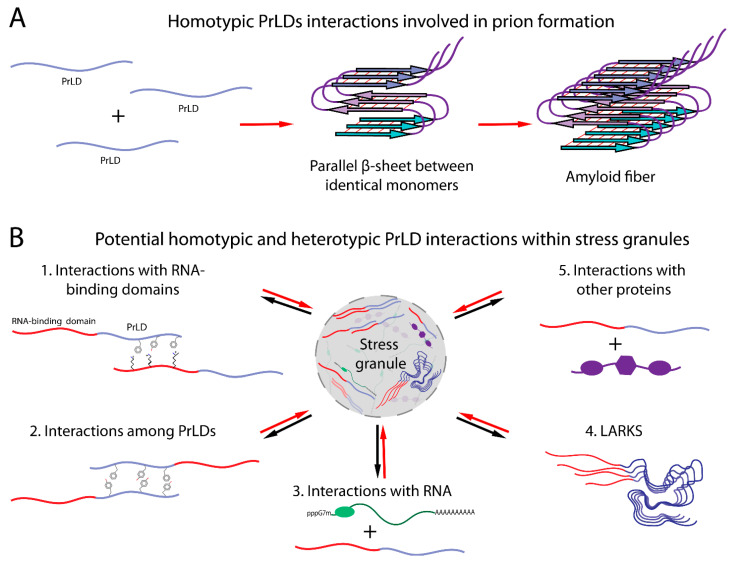
Potential PrLD interactions in amyloid fibers or within stress granules. (**A**) Upon prion formation, PrLDs assemble in an in-register cross-β structure involving homotypic interactions between identical monomers. (**B**) In stress granules, PrLDs have been proposed to engage in a variety of interactions, including homotypic interactions with other PrLDs, interactions with their corresponding RNA-binding domains, interactions with other stress granule proteins and RNA, and cross-β low-complexity aromatic-rich kinked segments (LARKS).

Although both prions and stress granules are macromolecular assemblies, the physical properties and complexity of these assemblies differ substantially. The material state of an assembly is dependent on the valence, strength, and duration of underlying interactions. Amyloid fibrils are homopolymeric solids involving highly stable interactions between identical or nearly identical proteins (Figure 1A). By contrast, stress granules contain a complex mixture of RNAs and proteins and are thought to involve dynamic multivalent interactions among the protein and RNA constituents (Figure 1B).

Because the interactions underlying stress granule recruitment and prion formation by PrLDs are fundamentally different, the amino acid sequence requirements for these two activities should likewise differ. Studies of the sequence and compositional determinants of prion propensity have provided insight into the mechanisms of formation and the structure of prions, and vice versa. Similar studies have begun to define how the sequence and composition of stress granule-associated PrLDs affect stress granule recruitment and dynamics. In this review, we use yeast prion proteins and stress granule-associated PrLDs as paradigms to examine how the sequence and composition of PrLDs affect the physical properties and phase behavior of protein assemblies. Understanding this relationship between PrLD sequence and phase behavior will be valuable in defining how cells form and regulate beneficial protein assemblies while preventing pathological aggregation.

## 2. Yeast Prion Sequence and Structure

**Figure 2:**
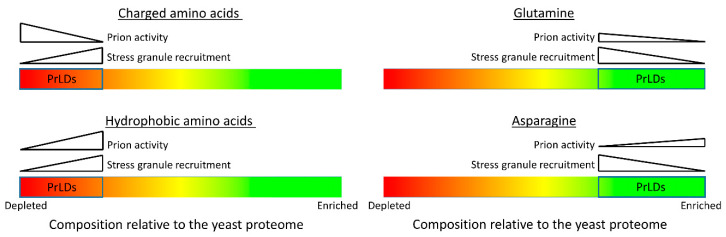
Compositional biases observed in PrLDs. PrLDs show strong compositional biases, including an enrichment in glutamine and asparagine, and a relative lack of charged and hydrophobic amino acids compared with the average of the yeast proteome. In yeast, screens of PrLDs have shown that different compositional features of PrLDs are associated with prion propensity [10,58] versus stress granule recruitment [73]. It is important to note that this figure represents general trends that were observed in these screens and, therefore, may not reflect the behavior of all PrLDs.

In 1994, Reed Wickner proposed that [*PSI*^+^] and [*URE3*], two previously identified nonchromosomal genetic elements, were the prion forms of Ure2 and Sup35, respectively [3]. Sup35 and Ure2 each contain an N-terminal prion domain that is responsible for prion formation [35,36]. These prion domains share similar compositional features, including enrichment in glutamine and asparagine, and relatively few charged and hydrophobic amino acids (Figure 2) [37]. In subsequent years, a number of other yeast prion proteins containing prion domains with similar compositional features have been identified [10,38,39,40,41,42,43]. However, it should be noted that not all amyloid-based prion proteins contain a PrLD: the mammalian prion protein PrP [2], the HETs prion protein from *Podospora anserina* [4], and the yeast prion protein Mod5 [44] each lack a glutamine/asparagine-rich PrLD. Likewise, not all prions are amyloid based [45,46,47,48].

Shortly after the discovery of the prion nature of [*PSI*^+^] and [*URE3*], it was recognized that the Ure2 and Sup35 prion domains could form amyloid fibrils [49,50], suggesting a likely mechanism for prion formation. Amyloid fibrils are filamentous cross-β structures, meaning that the β-strands run perpendicular to the fibril axis (Figure 1A). The individual β-strands within an amyloid fibril can arrange in a parallel or antiparallel manner, or the peptides can assemble as a β-helix. Studies demonstrating that prion formation by Ure2 and Sup35 is insensitive to prion domain scrambling [51,52] provided a first hint as to which architecture the proteins are adopting. Both antiparallel and β-helical structures are stabilized by complementary interactions between distinct residues in the sequence, so these interactions should be highly sensitive to scrambling. By contrast, in an in-register parallel β-sheet, the primary interactions are between identical residues among distinct protein monomers; thus, the same interactions are maintained after scrambling. Therefore, based on scrambling experiments, it was proposed that Ure2 and Sup35 adopt an in-register parallel β-sheet structure in their prion forms (Figure 1A) [53]. This prediction was subsequently supported by solid-state NMR experiments [54,55].

An in-register parallel β-sheet structure helps explain other compositional features that are associated with the propensity of PrLDs to form prions. The addition of charged residues into the core of a prion domain strongly inhibits prion formation [56,57,58], while the addition of hydrophobic residues strongly enhances prion propensity [59]. Both of these observations are consistent with an in-register parallel β-sheet structure, as stacked identical hydrophobic or charged residues will create stabilizing or repelling interactions, respectively. Indeed, the aggregation propensity of each amino acid can be predicted with reasonable accuracy based solely on hydrophobicity, charge, and β-sheet propensity [58,60].

Although scrambled Ure2 and Sup35 prion domains maintain the ability to form prions, they do so with efficiencies that are different from those of their wild-type counterparts [51,52], suggesting that while composition is the dominant determinant of prion propensity, primary sequence also plays a role. These modest primary sequence effects are also consistent with an in-register structure. The widths of amyloid fibrils formed by Ure2 and Sup35 are too narrow for the entire prion domains to be accommodated in a single long straight β-strand [49,50]; instead, the prion domains likely adopt a serpentine structure [61]. This structure may involve β-arches consisting of multiple β-strands separated by turns, as has recently been reported for the PrLD Orb2 [62], or more complex serpentine structures, analogous to the “Greek key” structure proposed for α-synuclein [63]. The sidechains from adjacent strands in these structures pack tightly to form a steric zipper [64], and the efficiency of this packing should be sensitive to primary sequence. Indeed, while prion propensity can be predicted with reasonable accuracy based solely on amino acid composition [12,14,15,58], methods designed to predict prion propensity that incorporate primary sequence [65], or explicitly model-predicted efficiency of packing between adjacent β-strands [66,67], may modestly improve prion prediction accuracy.

Surprisingly, there appears to be little correlation between the aggregation propensity of each of the amino acids and their prevalence in prion domains (Figure 2). Despite strongly promoting prion formation [59], hydrophobic amino acids are rare in yeast prion domains. By contrast, although yeast prion domains are dominated by polar amino acids, insertions of individual polar amino acids has little effect on prion propensity [58,68]. Mechanistic and modeling studies have helped explain this apparent contradiction. Yeast prion domains tend to be intrinsically disordered, and this disorder is thought to be important for prion formation [69]. Because these domains are disordered, a stickers-and-spacers model derived from the field of associative polymers [70,71], in which stickers are sites of noncovalent interaction, while spacers are the segments between stickers, is helpful in considering their assembly. Rare hydrophobic and aromatic amino acids act as the stickers, generally acting as the primary drivers of assembly. Polar amino acids act as disordered spacers and thus constitute the majority of the prion domains. However, although hydrophobic and aromatic amino acids contribute more to prion propensity on a per-residue basis, the spacers do also affect prion formation. While various polar and charged amino acids promote intrinsic disorder to varying degrees, and could thus act as disordered spacers, they have very different prion propensities; charged residues strongly inhibit prion formation, while glutamines and asparagines modestly promote prion formation [58]. Indeed, although individual glutamines or asparagines only weakly contribute to prion propensity, when present at sufficient levels, they can collectively act as significant contributors, as evidenced by the fact that extended polyglutamine tracts are sufficient for protein aggregation. Thus, the prevalence of glutamine and asparagine in prion domains is likely a result of their dual characteristics, promoting intrinsic disorder while also contributing modestly to prion propensity.

Furthermore, while many yeast prions are glutamine rich, among PrLDs, glutamine content is actually inversely correlated with prion propensity (Figure 2) [10,72]. This is likely because the identity of the spacer residues can also affect the material properties of the assemblies formed by PrLDs [71,72]. In particular, asparagine promotes the conversion to an amyloid state, while glutamine tends to promote the formation of nonamyloid assemblies [72]. Thus, even though glutamine is capable of promoting aggregation, asparagine is the preferred spacer residue among prion-prone PrLDs, likely because asparagine is more conducive to amyloid formation.

## 3. Cellular Interactions Influence the Sequence Requirements for Prions

While many of the compositional and sequence features observed in yeast prion domains can be rationalized based on intrinsic amyloid propensity, numerous chaperones and other cellular proteins affect yeast prion formation and propagation [74,75], and interactions with this cellular machinery also appear to impart sequence constraints on prion proteins. One example of these constraints has emerged from studies of interactions between Sup35 and the chaperone Hsp104. Maintenance of prions over multiple rounds of cell division requires the continual generation of new aggregate seeds (or propagons) to offset dilution by cell division. In yeast, the chaperone Hsp104 facilitates prion maintenance by cleaving prion aggregates into smaller fragments, thereby generating new propagons [76,77,78,79,80]. The Sup35 prion domain contains two distinct subdomains: a nucleation domain that is primarily responsible for the nucleation and growth of prion aggregates and an oligopeptide repeat domain that is largely dispensable for nucleation but required for prion maintenance [81]. These two subdomains have distinct compositional requirements. Specifically, while both hydrophobic and aromatic residues promote prion formation, only aromatic residues appear to promote prion maintenance [82]. Insertion of aromatic residues into polyglutamine segments reduces the average aggregate size in yeast [83], suggesting that aromatic residues promote chaperone-dependent aggregate cleavage. Interestingly, regions outside of the Sup35 prion domain also appear to be involved in Hsp104 binding [84], and the in-register parallel β-sheet structure likewise extends beyond the prion domain to varying degrees for different prion variants [85,86,87], highlighting that delineations between prion domains and nonprion domains are not absolute. While similar domains promoting chaperone-dependent prion propagation have not been as clearly defined in other prion proteins, the importance of chaperone-dependent fiber cleavage may be one reason why aromatic amino acids are more common than aliphatic amino acids in prion domains, despite the fact that both promote prion aggregation.

Interactions with the cellular proteostasis degradation machinery may also impose constraints on the sequences of yeast prion domains. Because protein aggregation is frequently deleterious, cells possess extensive proteostasis machinery design to prevent protein aggregation, including pathways to recognize and degrade aggregation-prone proteins [88,89,90]. Thus, for a protein to act as a prion, it needs to evade this machinery. The identities of both the stickers and the spacers in yeast prion domains appear to influence PrLD degradation by the proteostasis machinery, with both high glutamine/asparagine content and a paucity of aliphatic amino acids associated with lower rates of proteostatic degradation [91]. Likewise, for Sup35, a proteolytic cleavage event in the prion domain suppresses prion formation [92], highlighting that proteolysis can be used to regulate prion formation and/or propagation and may therefore impose additional sequence constraints on specific prion proteins.

## 4. Stress Granules

Despite the growing number of prions identified in yeast, the majority of yeast PrLDs do not appear to form prions [10]. Furthermore, it seems unlikely that most mammalian PrLDs would have evolved to form solid-phase prion-like aggregates. The majority of human PrLDs are found in RNA-binding proteins [16], and the aggregation of a number of these has been linked to degenerative diseases [93]. A possible alternative function for PrLDs has emerged from studies of RNP granules. Eukaryotic cells utilize compartmentalization strategies in order to maintain spatial control over biological processes. In addition to traditional membrane-bound compartments, such as the nucleus, cells contain membraneless compartments. These include various RNP granules, such as Cajal bodies [94], nucleolus [95], processing bodies (P-bodies [96]), germ granules [97], and stress granules [98]. These RNP granules vary in morphology, physical properties, localization, and physiological functions, but each is involved in the control of mRNA metabolism and localization.

Stress granules in particular have emerged as a useful paradigm for understanding the functions of nonprion PrLDs. During stress, eukaryotic cells undergo translational reprogramming, where mRNAs stalled in translation initiation (together with RNA-binding proteins and various other proteins and RNAs) are rapidly assembled into cytoplasmic stress granules [99,100]. Stress granules are dynamic and reversible; after the stress is relieved, stress granules disassemble, and mRNA can be returned into the translation pool or be targeted for degradation [99].

Super-resolution microscopy and fluorescence in situ hybridization experiments suggest that mammalian stress granules structurally consist of a dense inner core of mRNA–protein complexes, surrounded by a less concentrated shell layer [101]. Fluorescence recovery after photobleaching experiments suggests that the shell layer of stress granules engages in the rapid transfer and exchange of the components with the cytoplasm or P-bodies, while the core layer is significantly less dynamic [101]. However, the extent of stress granule dynamics seems to vary among organisms. While mammalian stress granules show liquid-like behavior, yeast stress granules have more viscous solid-like properties [102]. The material properties of stress granules may also change over time, becoming less liquid-like [103,104,105].

Core stress granule components can be purified biochemically; hundreds of proteins and thousands of RNA molecules have been identified within human stress granules or P-bodies to date [101,106]. Purification of the shell layer is more challenging, but proximity-labeling experiments have recently been used to catalog shell components [107,108]. Several RNA-binding proteins have been identified as primary components of stress granules, including TIA1 [22], PRRC2C [109], CSDE1 [109], UBAP2L [108], G3BP1 [110,111,112,113,114], and G3BP2 [110,111,112,113,114] in mammalian cells. Deletion or overexpression of these proteins significantly affects the size, number, and persistence of stress granules [109]. Additionally, the stress granule proteome includes translation initiation factors (eIF2, eIF3, eIF4A, eIF4B, eIF4G, and eIF4E), small 40S ribosomal subunits, poly(A)-binding protein (PABP), mRNA-degrading proteins, RNA helicases, and cell signaling factors [115,116].

Stress granules are thought to form in part through a process of liquid–liquid phase separation (LLPS). It has long been recognized that under certain in vitro conditions, many proteins will undergo liquid–liquid phase separation [117]. Moreover, RNA molecules can undergo LLPS in vitro, even in the absence of protein [118,119]. A growing body of evidence suggests that LLPS in vivo is involved in the formation of a wide variety of membraneless organelles [120]. LLPS occurs when a solution de-mixes into two liquid phases: one that is enriched for specific macromolecules and another that is depleted for these macromolecules [121,122]. The formation of an RNP by LLPS results in dense-phase droplets enriched in nucleic acids and proteins, surrounded by a diluted phase. The material state of this dense phase is determined by the valence, strength, and duration of underlying interactions, with weak and transient interactions allowing the dense phase to maintain a liquid character, as well as a dynamic exchange with the surrounding phase. Thus, stress granules can rapidly assemble in response to stress conditions; release their protein or mRNA components into the cytoplasm; and undergo docking, fusion, maturation, and exchange with other stress granules or RNP granules [99].

The biomolecular condensation of stress granules is promoted by a network of protein–protein, protein–RNA, and RNA–RNA interactions [120,122]. Many stress granule proteins are multivalent, allowing them to participate in multiple protein–protein and protein–RNA interactions involved in the formation or stabilization of stress granules. Although many protein–protein interactions occur between folded domains, intrinsically disordered regions (IDRs), including PrLDs, provide sites for interaction in many stress granule proteins.

Multiple lines of evidence suggest that PrLDs contribute to stress granule assembly or recruitment. The PrLD of the mammalian stress granule protein TIA-1 is required for efficient stress granule formation, and the prion domain of yeast Sup35 protein can substitute for the TIA-1 PrLD in supporting stress granule recruitment [22]. Other PrLDs have been similarly linked to the formation of various other membraneless organelles [123,124,125]. Additionally, in both yeast and mammalian cells, various PrLDs are actually sufficient for recruitment to stress granules [26,73,126,127].

## 5. Possible Mechanisms of PrLD Assembly in Stress Granules

A variety of mechanisms have been proposed by which PrLDs could contribute to stress granule assembly (Figure 1B). At high concentrations, the PrLDs of both FUS and hnRNPA2 form hydrogels [128]. While these gels are composed of cross-β amyloid-like structures, they are far more labile than those formed by yeast prion proteins [128]. This led Kato et al. to propose that these labile amyloid-like structures are key drivers of assembly in vivo. One challenge with this hydrogel model is that despite the labile nature of these hydrogels, they are less dynamic than mammalian stress granules. For example, in FRAP experiments, hnRNPA1 in stress granules shows rapid exchange with the cytoplasm, with fluorescence recovering in just a few seconds; by contrast, hnRNPA1 in hydrogels shows little recovery over this timeframe [126]. However, it is possible that cross-β interactions among a few molecules at a time could provide the more dynamic interactions that underlie stress granule formation [129].

A potential alternative mechanism of assembly was suggested by the discovery that many of the RNA-binding proteins found in stress granules can undergo LLPS [103,126,130]. In many cases, the PrLDs are sufficient for LLPS [103,126,130]. This LLPS is driven by a network of weak interactions between PrLDs. However, while isolated PrLDs are able to undergo LLPS in vitro, the relevance of this homotypic LLPS to stress granule formation in vivo is still debated. In vitro phase separation by isolated PrLDs frequently requires concentrations much higher than physiological concentrations. One possibility is that PrLDs provide weak, promiscuous interactions that act synergistically with more specific interactions to drive granule assembly [131]. Indeed, when the FUS PrLD is fused to light-activated oligomerization domains, induction of oligomerization can create nucleation centers with high local PrLD concentration, which can capture free PrLDs, resulting in localized LLPS [132].

Alternatively, the homotypic interactions that drive LLPS by isolated PrLDs may not be the principal driver of assembly for full-length proteins [133]. PrLDs could theoretically provide a variety of interactions that may contribute to stress granule assembly (Figure 1B). A study examining the FUS family of proteins showed that interactions between tyrosines in the PrLD and arginines in the RNA-binding domain drive FUS phase separation [71]. PrLDs could also contribute to RNA binding [126] or provide short linear motifs (SLiMs) that act as binding sites for interactions with other proteins [134].

Finally, in some cases, PrLDs may act as modulators of phase separation rather than drivers [135]. For example, deletion of a PrLD in the yeast stress granule protein Pub1 accelerates Pub1 condensation in response to heat stress and slows dissolution after heat stress, suggesting that the PrLD plays a role in protein solubilization [27]. Likewise, the yeast stress granule protein Ded1 forms heat-induced condensates at lower temperatures when its PrLD is deleted [28]. Finally, the yeast prion protein Sup35 undergoes pH-dependent phase separation in response to stress but forms more stable aggregates when the prion domain is deleted [135,136]. This suggests that some PrLDs may be involved in modulating the material state of stress-induced assemblies, increasing solubility or promoting reversible phase transitions, rather than driving assembly.

It is important to note that these possibilities are not mutually exclusive. The fact that various PrLDs are necessary [22,137] and/or sufficient [73,126] for stress granule recruitment suggests a direct role for some PrLDs in promoting the recruitment of their respective proteins to stress granules. However, these studies do not indicate whether these PrLDs engage in heterotypic or homotypic interactions within stress granules. Additionally, many PrLDs are not efficiently recruited to stress granules [73]; this, combined with the fact that in some cases PrLD deletion actually enhances stress granule recruitment, suggests that these PrLDs may act more as modulators of phase behavior. Finally, some PrLDs may do both, promoting recruitment to stress granules while also helping to maintain a liquid-like state.

## 6. Sequence and Compositional Features Promoting PrLD Recruitment to Stress Granules

Extensive efforts have been made to dissect how the sequences of PrLDs contribute to both LLPS and recruitment to membraneless organelles, such as stress granules. However, these efforts have been complicated by a lack of mechanistic understanding of how PrLDs contribute to the formation of membraneless organelles. Thus, parallel efforts have examined how PrLD sequence contributes to the cross-β interactions that underlie labile amyloid-like gels, as well as to other homotypic and heterotypic interactions that can promote LLPS.

Structural studies of gels formed by FUS [138,139,140] help explain the labile nature of these cross-β structures. While amyloid fibrils are typically stabilized by the interdigitation of extended β-strands to form a tight steric zipper [64,140], FUS amyloid-like assemblies involve highly kinked β-sheets; these kinks interfere with interdigitation, so instead, the β-sheets interact more weakly through hydrogen bonding and van der Waals interactions of polar and aromatic sidechains [138,139,140]. Stacking of aromatic amino acids appears to provide stability to the β-sheets [138]. This structural analysis has led to computational models to identify segments capable of forming kinked β-sheets, termed LARKS (low-complexity aromatic-rich kinked segments); LARKS are enriched in PrLDs found in membraneless organelles [138], suggesting a possible role for transient cross-β interactions in stabilizing these organelles (Figure 1B). Many of these proteins contain multiple LARKS, potentially providing multivalent interactions that could promote gel formation [138].

Other studies have sought to define the sequence features that drive LLPS by PrLDs and other IDRs. The driving force for LLPS is generally described using the stickers-and-spacers model discussed previously [70,71]. The valence (number) of stickers, the strength of the interactions between stickers, and the spacing between stickers can all influence LLPS propensity [141]. Various studies have indicated that π–π [142,143] and cation–π [71,144,145] interactions act as stickers and are key drivers of LLPS. Tyr, Phe, Trp, Asn, Glu, His, Gln, Asp, and Arg all have sidechains that can engage in π–π bonds; additionally, small amino acids, such as glycine, have exposed π orbitals in their peptide backbone amide groups [142]. Residues that are prone to engaging in π–π interactions are overrepresented in the PrLDs of stress granule-associated proteins [142]. In particular, uniformly spaced aromatic amino acids in PrLDs have been proposed to promote LLPS, while inhibiting the formation of solid-phase aggregates [141]. Additionally, the aromatic and positively charged amino acids can engage in cation–π interactions.

Charge–charge interactions have also been implicated in LLPS by IDRs. In particular, asymmetric charge distribution, resulting in patches of like charge, has been shown to promote phase separation by IDRs [146,147]. This may be a less significant driver of LLPS by PrLDs, which tend to have fewer charged residues than other IDRs [10]. However, it is worth noting that in a recent screen of PrLDs, higher content of charged residues was positively correlated with recruitment to stress granules, although the degree of asymmetric charge distribution did not show a significant correlation [73].

A recent examination of FUS highlighted how these different features can contribute to LLPS [71]. Cation–π interactions between tyrosines in the PrLD and arginines in the RNA-binding domain were found to be key drivers of LLPS [71], consistent with the findings of previous studies [144]. Other pairs of aromatic and positively charged residues could likewise promote LLPS, but less efficiently. Polar residues, such as serine, glutamine, and glycine, in the FUS IDR acted as spacers, but glycine helped maintain the fluidity of FUS assemblies, while glutamine and serine promoted hardening. Importantly, at least some of these results seem to apply in a cellular context, as the numbers of arginines [71] and tyrosines [71,132] in FUS affect LLPS propensity in cells. Interestingly, while tyrosine–arginine interactions were the dominant driving force for the assembly of full-length FUS, π–π interactions between tyrosine residues seemed to drive LLPS by the isolated PrLDs, albeit at significantly higher concentrations. Additionally, insertion of negatively charged residues into the PrLD promoted the phase separation of the PrLD with the RNA-binding domain but inhibited the assembly of the isolated PrLDs.

Collectively, these studies have identified various features that promote the assembly of some PrLD-containing proteins, leading to some progress in predicting the relationship between sequence and phase separation [141,142,143]. However, they have not yet yielded a generalizable set of rules that can accurately predict whether a given PrLD will be recruited to stress granules or other membraneless organelles. Part of the problem may be that because LLPS in cells is likely driven by a complex network of homotypic and heterotypic interactions, the interactions that promote LLPS by isolated domains or proteins in vitro may be different from the interactions that drive assembly in a complex cellular environment.

As an alternative approach, in a recent study, a set of PrLDs was screened for recruitment into yeast stress granules; the data set was then analyzed to identify common features associated with stress granule recruitment [73]. About a third of the tested PrLDs were sufficient to be recruited into granules. These PrLDs tended to be enriched in charged, aromatic, and hydrophobic amino acids, while showing no significant bias in protein net charge. The compositional biases observed among stress granule-recruited PrLDs were sufficient to predict whether other PrLDs would localize to yeast stress granules and to enable the design of synthetic PrLDs that reversibly localized to stress granules; additionally, scrambling of stress granule-recruited PrLDs did not block the recruitment, suggesting that the amino acid composition of these domains is more important than the primary sequence. PrLDs tended to respond similarly to heat stress, oxidative stress, and pH stress, suggesting a common mechanism of recruitment.

While these types of screens have the advantage of examining diverse PrLDs in cells, they have the limitation that they do not explain mechanistically how different sequence features contribute to the assembly. However, the sequence features that were associated with stress granule recruitment give some hints about the underlying interactions. The fact that sequence scrambling did not block stress granule recruitment suggests that specific interactions with primary sequence motifs are not a dominant driver of recruitment. This is consistent with the findings of a previous study showing that simple SYGQ repeats are sufficient to promote stress granule recruitment in mammalian cells [148], and demonstrates that rather generic compositional features can nonetheless lead to specific targeting. The strong enrichment of charged residues suggests that the formation of labile amyloid-like gels is likely not a principal driver of recruitment for these PrLDs, as charged residues should disrupt the in-register parallel β-sheet interactions thought to underlie these gels [138,139,140]. However, it remains possible that a subset of PrLDs may form amyloid-like gels, or that while initial recruitment is driven by LLPS (thus explaining the bias towards charged residues), gel-like interactions may form after recruitment, as stress granules mature. Likewise, it is possible that only a small fraction of each PrLD engages in cross-β interactions, and that charged residues are accommodated outside of this region. Thus, additional experiments will be required to fully define the mechanistic basis for these observations. Additionally, further experiments will be required to determine whether similar features drive stress granule recruitment in mammalian cells.

Finally, it is important to note that because different PrLDs are likely recruited to stress granules by distinct mechanisms, the general trends that have emerged from screens of stress granule-associated PrLDs, or from studies of individual PrLDs, may not apply to all PrLDs. A recent study examining phase separation by components of hnRNPA2-containing transport granules highlights this challenge [149]. In these experiments, hnRNPF, a PrLD-containing protein found in hnRNPA2 granules, did not efficiently phase-separate on its own. However, it partitioned into droplets formed by the hnRNPA2 low-complexity domain (LCD), but not the FUS LCD. Arginine residues in the hnRNPA2 LCD were critical for its co-phase separation with hnRNPF. Interestingly, ch-TOG, another hnRNPA2 granule component, also specifically phase-separated with the hnRNPA2 LCD but engaged in a distinct set of interactions with the hnRNPA2 LCD. While these results provide a nice illustration of how the specificity of targeting to membraneless organelles can be achieved, they also demonstrate the challenges of predicting targeting to complex organelles, such as stress granules, where multiple interactions may contribute to the recruitment, and where different proteins may be recruited by distinct mechanisms. PrLDs that act as scaffolds for nucleating assembly likely have compositional requirements that are different from client proteins that subsequently partition into these assemblies. Additionally, even among scaffolds and clients, there are likely various potential mechanisms of assembly or partitioning, each of which may have distinct compositional requirements.

## 7. Distinct Classes of Prion-Like Domains

Our current methods for identifying PrLDs were developed based on studies of yeast prions, making it remarkable that these methods (particularly PLAAC) are so effective at identifying mammalian disease-associated PrLDs. When the human proteome is scanned with PLAAC, among the 10 highest scoring human proteins that contain an RNA recognition motif, mutations in more than half of these proteins have been linked to degenerative diseases [25]. Additionally, methods derived from studies of yeast prion domains have been effective at predicting the effects of some disease-associated mutations in PrLDs [33,150]. This suggests that at least in some cases, the sequence features driving prion formation overlap with those associated with pathological assembly.

However, while these methods have aided in the identification of some disease-associated PrLDs, they do not appear to be broadly effective at predicting whether a PrLD will be recruited to stress granules. Among yeast PrLDs, stress granule recruitment is actually inversely correlated with PLAAC scores [73]. This reflects differences in the compositional features that promote prion formation versus stress granule recruitment (Figure 2). For example, charged residues inhibit prion formation [57,58] but are positively correlated with stress granule recruitment [73]. Likewise, high asparagine content [72] is correlated with amyloid propensity for PrLDs, while it is negatively correlated with stress granule recruitment [73]. Given that fundamentally different interactions drive prion formation versus stress granule recruitment, it is unsurprising that different compositional features would promote each form of assembly; however, these differences highlight the limitations of using methods derived from the analysis of yeast prions to predict nonprion assembly. Further refining our understanding of the compositional features that promote different types of PrLD assemblies may aid in the development of more targeted prediction methods.

This observation also suggests that it may be a mistake to view PrLDs as a single homogenous class. The term “prion-like domain” is not a functional definition based on actual prion-like activity; it is a descriptive term based on compositional similarity to proteins with prion-like activity. Some PrLDs have clear amyloid or prion-like activity, but many do not [10,151], just as some PrLDs promote recruitment to stress granules [22,26,73], while others inhibit recruitment [27,28]. Furthermore, it is striking that many of the major compositional biases that promote recruitment to yeast stress granules actually make the domains less “prion-like” (Figure 2). While PrLDs are generally enriched in polar amino acids, and depleted in charged and hydrophobic residues, PrLDs that are recruited to stress granules tend to have less extreme versions of each of these biases, although they still have higher polar content and lower charged and hydrophobic content than the average in the yeast proteome [73]. It is unclear how far these trends can be extrapolated. Many IDRs that are outside of the traditional definition of “prion-like” composition are also thought to contribute to the assembly of stress granules and other membraneless organelles, so it is likely that some of the lessons learned from the studies of PrLDs may extend to these other IDRs.

## 8. Stress Granule Regulation and Dysregulation

**Figure 3:**
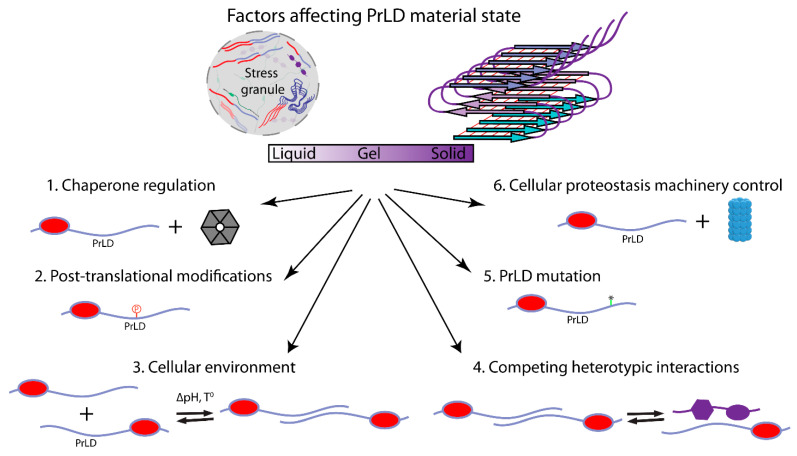
Some of the key factors that affect interactions and the material state of PrLDs in cells. Post-translational modifications and PrLD mutation can affect both intrinsic assembly propensity and interactions with other partners. Changes in the cellular environment can directly alter the assembly propensity of PrLDs or alter regulatory interactions. Changes in the concentration or localization of PrLDs and their binding partners can modulate their assembly propensity. Chaperones and cellular proteostasis machinery affect the concentration of proteins and modulate the dynamics of interactions.

Studies of the interactions that facilitate PrLD recruitment to stress granules provide some insight into possible mechanisms by which this recruitment may be regulated or perturbed (Figure 3). A variety of molecular chaperones have been linked to proper control of stress granule dynamics and to efficient dissolution or degradation of stress granules after stress [99]. Intriguingly, studies of FUS suggest that nuclear import receptors can act as a chaperone, preventing aberrant phase transitions by PrLD-containing RNA-binding proteins [152,153,154], likely by binding to regions involved in LLPS. RNA can also both positively and negatively affect assembly propensity. For example, low levels of RNA promote FUS LLPS in vitro, while high levels inhibit LLPS; likewise, in vivo, reduction of RNA levels promotes FUS LLPS, while an RNA known to have high binding affinity to FUS promotes assembly [155]. Other interacting partners may likewise positively or negatively modulate assembly propensity, by either masking assembly-prone domains or providing a scaffold for assembly.

Various post-translational modifications have also been shown to modulate LLPS by PrLDs [156,157]. These post-translational modifications can alter molecular interactions that regulate LLPS or can directly modify the ability of a PrLD to engage in the π–π, cation–π, and charge–charge interactions that are thought to drive PrLD LLPS. Arginine methylation in FUS, Ddx4, and hnRNPA2 inhibits LLPS and decreases protein accumulation inside stress granules due to a decrease in cation–π interactions [137,146,154]. Phosphorylation of serine or tyrosine residues, which are enriched in PrLDs, alters protein charge and changes protein steric and chemical properties. Depending on the context, these changes can promote charge–charge interactions or can create charge or steric repulsion. Thus, phosphorylation inhibits the LLPS of some PrLD-containing proteins, including FUS [158] and TDP-43 [159], while promoting LLPS for others, including TIAR-2 [160]. Other modifications, including lysine acetylation, citrullination, and O-linked GlcNAc modifications, within intrinsically disordered regions influence phase separation [156,157].

Additionally, changes in environmental conditions can directly affect the assembly properties of proteins containing PrLDs and other IDRs. For example, energy depletion in yeast results in a drop in cytoplasmic pH [161]. Phase separation by Sup35 is regulated by changes in the charge of its IDR in response to this pH change [136]. Likewise, heat-induced assembly of the yeast Pab1 protein appears to be a direct response to both elevated temperature and the resulting drop in cellular pH [162]. Interestingly, in both of these cases, low-complexity domains in the proteins seem to be modulators, rather than drivers, of assembly [163].

Finally, studies of PrLDs in stress granules have provided insight into how mutations or aberrant post-translational modifications in PrLDs can contribute to disease. Many disease-associated mutations are believed to alter the material state of stress granules, thus contributing to a dysregulation of RNA metabolism. In some cases, mutations promote the formation of stable, solid-phase amyloid-like aggregates by directly altering the assembly propensity of PrLDs [103,126]; this formation of solid-phase assemblies could theoretically occur either directly from dilute solution or within the context of LLPS assemblies. Indeed, mutations rationally designed to increase the aggregation propensity of hnRNPA2B1 are sufficient to cause pathology in a *Drosophila* model of multisystem proteinopathy [68]. PrLD mutations can also alter phase separation propensity [164] or modulate the dynamics of biomolecular condensates, thus altering the material properties and functions of the condensates [165].

Alternatively, rather than altering the intrinsic phase separation properties of a PrLD, PrLD mutations can perturb heterotypic interactions that modulate assembly propensity. The high local concentration of PrLDs in stress granules should increase the probability of aggregation. However, heterotypic interactions with binding partners, such as RNA or proteins, can compete with the homotypic interactions that nucleate aggregation; this phenomenon has been referred to as heterotypic buffering [165]. Thus, mutations that alter the relative concentrations of or interactions between stress granule components can also contribute to the aberrant assembly of PrLD-containing proteins or change the material properties of stress granules. Mislocalization can similarly contribute to aberrant assembly, in part by disrupting interactions that prevent assembly. For example, many of the ALS-associated mutations in FUS are in its nuclear localization signal [166]. Since RNA is thought to inhibit FUS assembly, the lower concentration of RNA in the cytoplasm may stimulate aberrant phase transitions [155]. Stress granules also contain high concentrations of chaperones that can prevent PrLD aggregation [167,168,169], and the autophagy [170] and ubiquitin–proteasome pathways [171,172] are linked to stress granule dynamics and clearance. Mutations in these pathways are correlated with abnormal stress granule dynamics.

## 9. Conclusions and Future Perspectives

Proteins containing PrLDs are capable of forming a variety of both functional and pathogenic protein assemblies. While significant progress has been made in defining sequence features that are responsible for prion formation, the sequence features driving other types of PrLD assemblies, and how sequence changes affect the material state of these assemblies, are less clear.

Likewise, many questions remain about the exact functions of PrLDs in stress granules and other membraneless organelles. While some PrLDs are necessary or sufficient for stress granule recruitment, others appear to act more as regulators of solubility, preventing the formation of stable protein aggregates and maintaining the dynamic nature of RNP assemblies. Preliminary progress has been made in defining the sequence features that promote PrLD recruitment to stress granules. However, the key interactions that PrLDs engage in within stress granules, the mechanisms by which assembly and disassembly are regulated, and the mechanisms by which PrLDs are targeted to specific membraneless organelles are still not fully understood.

Answering these questions may provide insight into both the normal functions of PrLDs and the mechanisms by which mutations in PrLDs lead to pathology, potentially leading to new targeted therapies for various devastating human neurodegenerative disorders.

## Data Availability

No new data were created or analyzed in this study. Data sharing is not applicable to this article.
